# The Last Straw: Conflicts and Suicide Attempts in Armenian Adolescents

**DOI:** 10.1192/j.eurpsy.2023.701

**Published:** 2023-07-19

**Authors:** M. Arsenyan

**Affiliations:** Republican Child Psychiatry Department, “St. Grigor Lusavorich” MC, Yerevan, Armenia

## Abstract

**Introduction:**

Adolescence is, arguably, the most vulnerable period of a person’s development when the susceptibility to emotional-affective and behavioural disorders is at its height and conflict situations can result in self-injuries and suicide attempts, some with lethal outcomes (Woycex W., Сlinical Suicidology, 2007.-280p). Research indicates that conflict with family is associated with suicide attempts in adolescents (Elise P. JAD, 2018; 241:499-504). There is a small body of research on the risk factors for suicide attempts in Armenian adolescents. However, there is a lack of research on understanding what role conflict can play in developing suicidal thoughts and behaviour.

**Objectives:**

The present research aims to understand the role of conflict in suicide attempts among Armenian adolescents.

**Methods:**

The researcher conducted a qualitative analysis of 39 patient histories of adolescents hospitalised after a suicide attempt using the documentary method. The patient histories included, among others, the results of psychiatric tests using Hamilton’s Rating Scale for Depression (HAM-D), Hamilton’s Rating Scale for Anxiety (HAM-A), Columbia Suicide Severity Rating Scale (C-SSRS), and the records of psychiatric consultation.

**Results:**

The psychiatric tests showed that all adolescents suffered either mild, moderate or severe levels of depression and anxiety. They also exhibited mild, moderate or severe suicide risk, based on C-SSRS. The results revealed that before the suicide attempt, adolescents were exposed to continuous distress and traumatic events at home that lasted for months, sometimes years. They often witnessed domestic abuse and attempted to protect one of the parents (usually their mother) from being physically and psychologically abused. In some cases, the distress was the result of parents trying to end the adolescent’s relationship with their boyfriend. During psychiatric consultation, adolescents disclosed reoccurring suicidal thoughts and suicidal ideation when exposed to continuous trauma and distress at home. In most cases, conflict with parents was the trigger that made adolescents act. The conflict was the last straw that pushed them to attempt suicide.

**Conclusions:**

Suicide attempt in Armenian adolescents remains one of the least investigated areas. Domestic abuse is often silenced and almost never reported to authorities. This usually leaves adolescents one-on-one with their struggles. The needs of adolescent witnesses of domestic abuse and those suffering controlling behaviour and developing suicidal thoughts, some of them eventually attempting suicide - remain largely unaddressed. More research is needed to understand factors associated with suicidal behaviour in Armenian adolescents. Research studies can hopefully become a basis for a future centralised mental health strategy aimed at helping adolescents find a way out and not resort to suicidal attempts.

**Disclosure of Interest:**

None Declared

